# Cellular based immunotherapy for primary liver cancer

**DOI:** 10.1186/s13046-021-02030-5

**Published:** 2021-08-09

**Authors:** Yuanyuan Zheng, Yan Li, Jiao Feng, Jingjing Li, Jie Ji, Liwei Wu, Qiang Yu, Weiqi Dai, Jianye Wu, Yingqun Zhou, Chuanyong Guo

**Affiliations:** 1 Department of Gastroenterology, Putuo People’s Hospital, Tongji University, Shanghai, 200060 China; 2 Department of Gastroenterology, Shanghai Tenth People’s Hospital, Tongji University School of Medicine, Shanghai, 200072 China

**Keywords:** Primary liver cancer, Cellular based immunotherapy, Lymphoid cell, Myeloid cell, Combined immunotherapy

## Abstract

Primary liver cancer (PLC) is a common malignancy with high morbidity and mortality. Poor prognosis and easy recurrence on PLC patients calls for optimizations of the current conventional treatments and the exploration of novel therapeutic strategies. For most malignancies, including PLC, immune cells play crucial roles in regulating tumor microenvironments and specifically recognizing tumor cells. Therefore, cellular based immunotherapy has its instinctive advantages in PLC therapy as a novel therapeutic strategy. From the active and passive immune perspectives, we introduced the cellular based immunotherapies for PLC in this review, covering both the lymphoid and myeloid cells. Then we briefly review the combined cellular immunotherapeutic approaches and the existing obstacles for PLC treatment.

## Background

The twentieth century recorded increased cancer mortality rates, of which primary liver cancer (PLC), the fourth most lethal carcinoma, accounted for 8.2% of the total [1, 2]. The disease has the highest incidence rate in eastern Asia, and globally, every year, approximately 841,000 new cases and 782,000 deaths are recorded [1]. Therefore, PLC is a serious health and economic burden.

Histologically, PLC primarily comprises hepatocellular carcinoma (HCC) (75-85%), intrahepatic cholangiocarcinoma (iCCA) (10-15%) and other rare cases [2]. With complex etiological variation and occult clinical features, PLC is predominantly diagnosed at stages not early enough for simple surgical resection treatment, and patients experiencing high recurrence rates [3–6]. Targeted treatments based on natural or synthetic drugs revealed the positive antitumor effects against PLC [7–10]. Further researches have shown that combined therapeutic approaches, including interventional therapy, radiotherapy, chemotherapy and biotherapy, improve the curative effects and the possibility for individual treatment in PLC [11]. HCCs and iCCAs potentially share the common hepatocyte origins despite their histologically distinctive clinical features, and a final tumor phenotype could be affected by interactions between the immune microenvironment and oncogenes [12]. Immune surveillance, whereby tumor cells are eliminated at nascent stages, protects the body from tumors. Using antigenic modulation, tumor-derived soluble factors, and immunological ignorance and tolerance strategies, tumor cells become capable to survive from the host’s immune attack, and homeostasis gradually proceeds from immune surveillance to equilibrium and further immune escape during tumor progression [13]. These interactive procedures suggest immunological intervention may have potential to limit or even reverse this phenotypic transformation, under certain conditions.

**Fig. 1:**
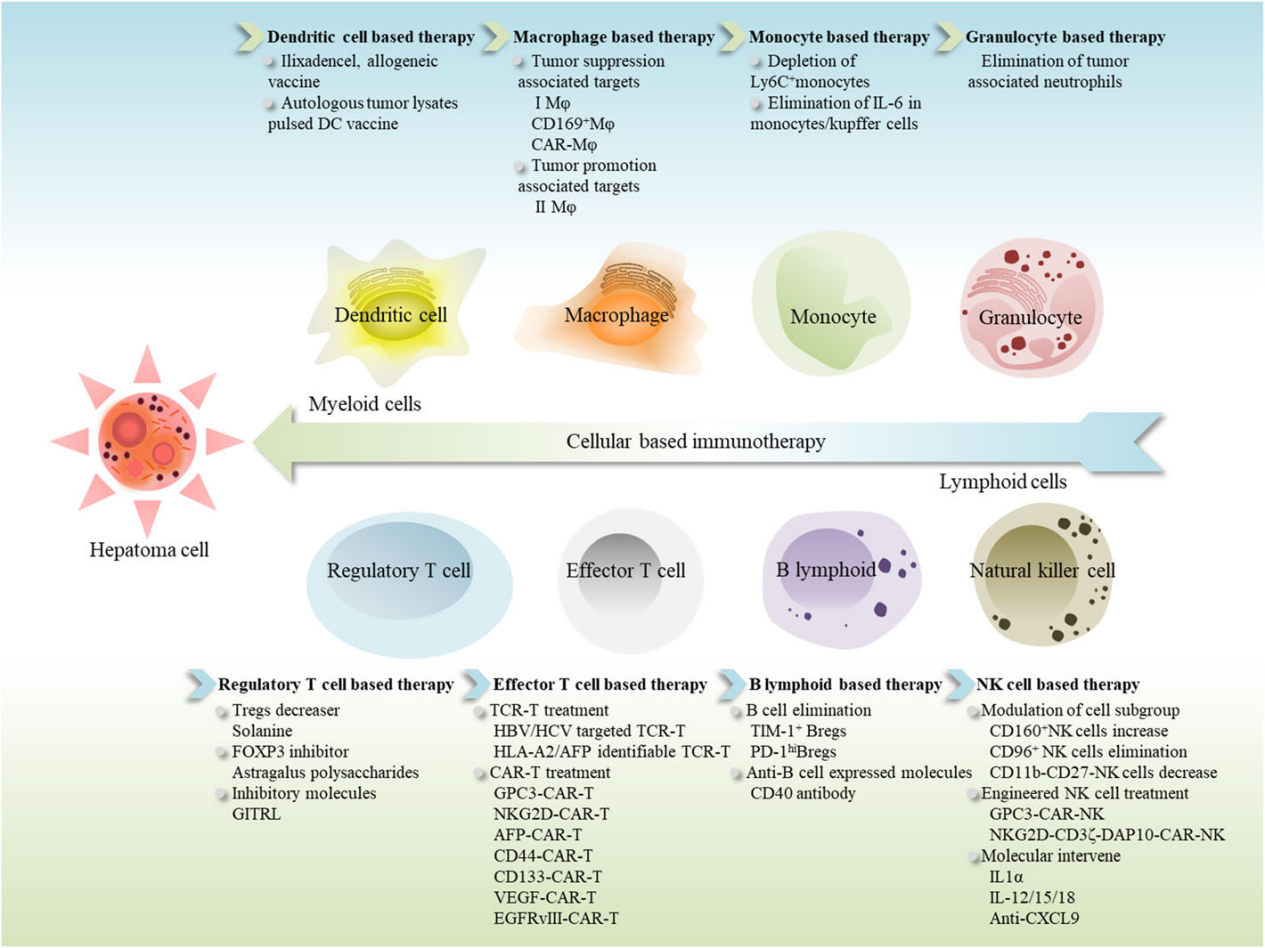
Cellular based immunotherapy in liver cancer. Based on myeloid or lymphoid immune cells, strategies are attempted for liver cancer therapy. In myeloid cell group, DC vaccine, engineered Mφ and depletion of immune suppressors are undergoing research for hepatoma treatment. For lymphoid cells, strategies such as T/NK cell engineering, Tregs/Bregs depletion and molecular regulatory intervenes are also under study. DC, dendritic cell; Mφ, macrophage; Tregs, regulatory T cells; Bregs, regulatory B cells; FOXP3, forkhead box protein P3; GITRL, ligand to Tregs evoked glucocorticoid induced tumor necrosis factor receptor; TCR-T, T cell receptor engineered T cells; CAR-T, chimeric antibody receptor engineered T Cells; HBV, hepatitis B virus; HCV, hepatitis C virus; HLA-A2, human leukocyte antigen-A2; AFP, A-fetoprotein; GPC3, Glypican-3; NKG2D, NK group 2 member D; VEGF, vascular endothelial growth factor; EGFRvIII, epidermal growth factor receptor variant III; TIM-1^+^, T cell immunoglobulin mucin domain-1 positive; PD-1, programmed cell death-1; CXCL9, chemokine C-X-C motif chemokine ligand-9; IL, interleukin; CD169, cluster of differentiation 169; CD44, cluster of differentiation 44; CD133, cluster of differentiation 133; CD40, cluster of differentiation 40; CD160, cluster of differentiation 160; CD96, cluster of differentiation 96; CD11b, cluster of differentiation 11b; CD27, cluster of differentiation 27; CD3, cluster of differentiation 3; DAP10, DNAX-activating protein 10

By modifying the immune system to elicit antitumor capabilities, immunotherapies are viable alternatives for PLC therapy. These aforementioned immunotherapeutic strategies can be categorized into active and passive immunotherapy according to the immune response mechanisms. Immune cells play crucial roles in the antitumor procedure constructed by immune system both in innate and adaptive immunity, thus cellular based immunotherapy has underpinned numerous immunotherapeutic approaches for PLC currently. Based on the means of interventions, the cellular immunotherapies are typically composed of active and passive immune therapeutic strategies. In this review, we introduce the cellular based immunotherapeutic approaches for PLC (Fig. 1), with a brief overview of combined cellular treatments and current therapeutic issues.

## Active cellular immunotherapy in primary liver cancer

### Cell vaccines

Vaccine treatments are based on tumor antigens which activate the host’s immune system to eliminate tumor cells and memorize abnormal antigens for tumor recurrence. Aimed at evoking immune response to tumor-specific/associated antigens (TSAs/TAAs), exogenous vectors, intracellular elements (peptides, proteins or nucleic acids), and correlated cells have been proposed as antitumor vaccines [14]. Cell vaccines, including allogeneic and autologous groups [15], arise several approaches for PLC therapy. When compared with viral, bacterial or yeast vectors, cell vaccines are advantageous of avoiding the immune responses triggered by exogenous vector carriers.

Allogeneic cell vaccines are usually prepared from tumor cells or lysate collections, with or without gene modification, before final TSA/TAA delivery to the immune system. The approach is advantageous as reagents can be mass produced but is flawed in terms of maturation for antigen presenting cells (APCs) [15, 16]. In vitro evidence suggested that the allogeneic cell lines, HepG2 and BEL7402, when co-cultured with autologous dendritic cells (DCs), the functional APCs in the body, emerged a positive activation of both CD4^+^ and CD8^+^ T cells against autologous hepatoma cells [17, 18]. An in vitro iCCA study revealed similar results to HCC: RNA and protein lysates extracted from iCCA cell lines have talents to pulse DCs and enhance T cell cytotoxicity against cholangiocarcinoma [19]. Clinical trials on allogeneic cell vaccines against PLC are also underway (Table 1) (clinicaltrials.gov). Based on antitumor immunity in animal models induced by allogeneic cancer stem cell vaccination [28], further clinical trials against HCC have been completed and awaiting results (NCT02089919). The phase I clinical trial of ilixadencel [20], an allogeneic DC vaccine, confirmed its safety and effectiveness in activating tumor specific immune responses in advanced HCC (NCT01974661). However, more in-depth investigations are required to apply these therapies to PLC in clinical settings.

**Table 1:** Clinical therapeutic trials for cell vaccines against PLC

Vaccine catalog	Trial Phase			Enrolled patients backgrounds			Trial information		
	I	II	III	Number.	Location	intervention	comments	Identifier	Reference
Allogeneic				18	Sweden	allogeneic dendritic cell vaccine (ilixadencel)	Safety; immunological response activated against HCC	NCT01974661	[20]
Autologous		I/IIa		8/12	China	autologous fixed HCC vaccine	Safety; recurrence delay for patients with HCC after operation	–	[21]
				41	China	autologous fixed HCC vaccine	Safety; recurrence rates reduced and overall survival rates improved for patients with HCC after operation	–	[22]
				8	USA	bi-shRNA^furin^/GM-CSF incorporated autologous HCC cell vaccine	Safety; immunological response activated against HCC and overall survival prolonged	–	[23]
	-			160	China	autologous DC-tumor vaccine	Safety; recurrence and metastasis rates for postoperative HCC patients reduced; survival rates improved	–	[24]
				10	Japan	autologous tumor lysates pulsed DC vaccine	Safety; antitumor efficiency probably existed against PLC: delayed type hypersensitivity induced (7/10); tumor size shrinked (1/10); serum level of tumor marker decreased (2/10)	–	[25]
				31	China	autologous tumor lysates pulsed DC vaccine	Safety; HCC patients’ survival better prolonged by boosters followed DCs therapy than single DCs vaccine itself	–	[26]
				36	Japan	autologous tumor lysates pulsed DC vaccine	Survival for iCCA patients prolonged; prognosis improved	UMIN000005820	[27]

Abbreviations for the table: *HCC* Hepatocellular carcinoma, *iCCA* Intrahepatic cholangiocarcinoma, *DCs* Dendritic cells, *GM-CSF* Granulocyte macrophage colony stimulating factor, *USA* United States of America

Autologous cell vaccines, which present effective TSAs/TAAs, are derived from and returned to patients after in vitro manipulation [15]. Both tumor cell based and APC based autologous vaccines have displayed anticancer potential towards PLC in recent studies (Table 1). The safety of hybrid cell vaccination was certified in liver involved metastatic melanoma [29]. The Hepa 1-6 cell vaccine, equipped with granulocyte macrophage colony stimulating factor (GM-CSF) and interleukin-2 (IL-2) as adjuvants, was protective against HCC in a syngeneic C67L/J mouse model, and the autologous fixed tumor formulation vaccine was validated as preventing HCC recurrence in phase I/II clinical trials [21, 22]. In other research, a bi-shRNA^furin^/GM-CSF incorporated autologous HCC cell vaccine, FANG™, stabilized PLC for over 4 months in five patients during a phase I trial, with four patients experiencing more than 2 years’ survival, which surpassed the 7.9 month median survival rate of sorafenib in a phase III trial [23, 30]. Autologous DC-tumor vaccines have also shown safety and protective effects from recurrence and metastasis for postoperative HCC [24]. The safety of tumor lysate pulsed DC vaccines for PLC was tentatively confirmed in other clinical trials, while boosters following DC therapy showed increased efficacy in prolonging survival for HCC patients when compared with single pulsed DC vaccines [25, 31]. A previous study showed that iCCA cell lysate pulsed autologous DCs, especially gene modified self-DCs, enhanced effector T cell cytotoxicity against iCCA [26]. A 6-year follow up based clinical trial on appraising the positive effects of tumor lysates pulsed DC vaccine for iCCA also demonstrated the feasibility and effectiveness (UMIN000005820) [27]. Preliminary findings on autologous cell vaccine for PLC therapy showed positive achieves, however, further investigations are still needed to better understanding the underlying mechanisms for clinical applications.

### Negative lymphoid regulatory cell blockage

Lymphoid regulatory cells participate in monitoring internal immune homeostasis, the negative functional ones including FOXP3^+^CD25^+^CD4^+^ regulatory T cells (Treg) and regulatory B cells (Breg) possess inhibitory roles on antitumor immunity in liver cancer [32–34]. Strategies to block regulatory cell mediated immunosuppression, either by depleting effector regulatory cells or modulating correlated activating pathways, may play crucial roles in achieving immunotherapy against liver cancer.

**Fig. 2:**
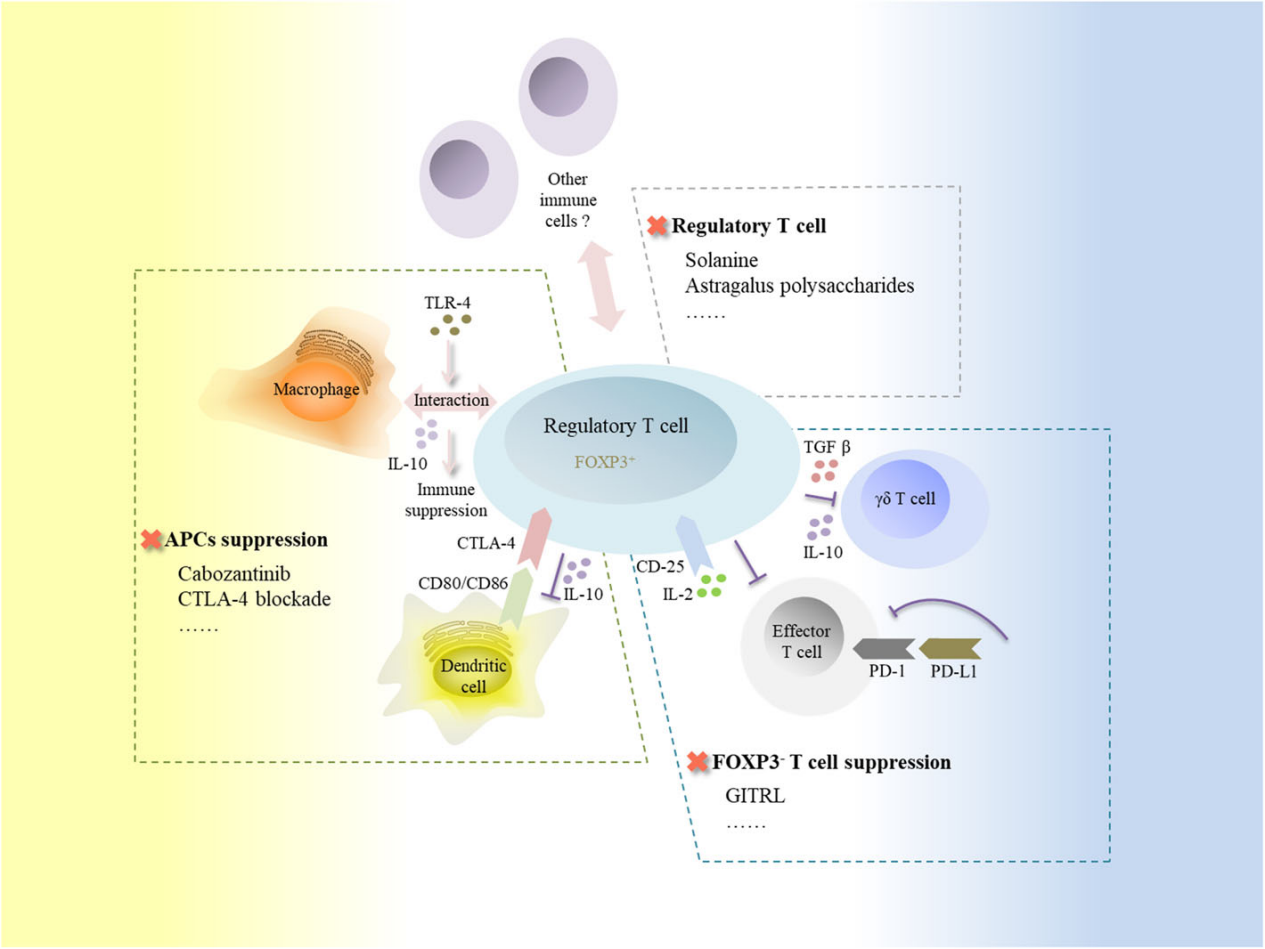
Tregs interact with immune cells and the therapies in liver cancer. Tregs suppress anti-liver cancer immunity via interacting with several immune cells. Firstly, Tregs inhibit APCs’ function in liver cancer, currently known mechanisms like CTLA-4 ligand expression to down-regulate DCs’ CD80/86 and IL-10 secretion to inhibit DCs maturation, TLR-4 signal mediated immune suppression with macrophage participant. And the APCs suppression may be rescued by drugs like cabozantinib and CTLA4 blockade. Secondly, Tregs suppress FOXP3^−^ T cells in liver cancer such as effector T cell (consuming IL-2 with highly expressed CD25; PD-1 correlated dysregulation) and γδ T cell (depending on TGF β and IL-10), which can be partially blocked by GITRL therapy. And regulatory T cell itself can be depressed by CD4^+^CD25^+^ Tregs’ proportion decreaser solanine and *FOXP3* expression inhibitor astragalus polysaccharides for liver cancer therapy. CTLA-4, cytotoxic T lymphocyte associated antigen-4; IL-2, interleukin-2; IL-10, interleukin-10; TLR-4, Toll like receptor-4; PD-1, programmed cell death-1; PD-L1, programmed cell death-ligand 1; TGF β, transforming growth factor β; GITRL, glucocorticoid induced tumor necrosis factor receptor ligand; CD25, cluster of differentiation 25

Tregs are broadly classified into thymus-derived naturally occurring Tregs (tT_reg_ cells) and peripherally derived induced Tregs (pT_reg_ cells), cohesively regulating internal immune homeostasis [35, 36]. Tregs suppress APC function via down-regulating CD80/CD86 with cytotoxic T lymphocyte associated antigen-4 (CTLA-4) expression, and reduce responder T cells by competitively consuming surrounding interleukin-2 (IL-2), as key mechanisms in cancer immune suppression, proceed effector T cell anergy in antitumor response [36]. The immune suppressive modulation of singularly recruited Tregs in PLC has been validated both in vitro and vivo studies [32, 37–40]. Currently, Tregs blunt antitumor immunity via immune cell correlated intervening approaches in PLC (Fig. 2): 1) APC suppression: Tregs harvested from HCC mice inhibit DC function by down-regulating the co-stimulator CD80/86 via CTLA-4 expression, secreting inhibitory cytokines such as IL-10 to weaken DC maturation and the tumor necrosis factor-α (TNF-α)/IL-12 production, and inhibiting via cell to cell contacts [41]. The toll like receptor-4 (TLR-4) triggers interactions between Tregs and macrophages, leading to immune suppression in HCC [42]. 2) FOXP3^−^ T cell suppression: Tregs attributed to the programmed cell death-1 (PD-1) correlated dysregulation of T cell population frequencies, with the exhaustion of functional T cells [40, 43]. Cytokines like IL-2 with its highly expressed CD25 receptor on Tregs may also play critical role in functional T cell toxicity [44, 45]. Tregs impair γδ T cells and down-regulate the interferon-γ (IFN-γ) secretion of γδ T cells in a transforming growth factor β (TGF-β) and IL-10 dependent manner [37]. Previously, the deletion of tumor infiltrating Tregs was demonstrated to enhance HCC specific immunotherapy [46]. Drugs such as solanine (CD4^+^CD25^+^ Tregs’ proportion decreaser) [47] and astragalus polysaccharides (*FOXP3* expression inhibitor) [48] also revealed antitumor enhancement in HCC via Treg suppression. Based on predecessor’s work, attempts on rectifying Tregs mediated immune dysregulation in PLC therapy are never stagnation. Tregs evoked glucocorticoid ligands induced tumor necrosis factor receptor expression in PLC, and are proposed as potential treatments for PLC by decreasing Treg immunosuppression and reactivating CD4^+^CD25^−^ T cells [32]. Furthermore, when combined with a CTLA-4 blockade, this was shown to improve antitumor efficacy during treatment [49]. For patients resistant to immune checkpoint inhibitor treatment, cabozantinib, exerted its immune regulation effects via releasing HGF (hepatocyte growth factor) correlated DCs suppression and Tregs promotion, is currently being explored in a phase III clinical trial to verify its therapeutic capacity for HCC (NCT04588051). Further exploration for the Treg based effector mechanisms and therapeutic methods are required in PLC.

Bregs, differentiated from B lymphoid cells, were demonstrated to play pivotal roles in anti-immune response activity against tumor, while their surface tags for phenotype classification have not yet reach consensus so far [50, 51]. To elicit immune suppressive efficacy during tumorigenesis, Bregs function in diverse differentiation and functional mechanism in immune cells, such as IL-10 dependent inhibition on APCs and cytotoxic T cells, and the cytokine (IL-10, TGF-β) relied promotion of FOXP3^+^ T cell differentiation in the immune system [50].

A high Breg frequency was correlated with HCC in rats [52], the correlation was also supported by the raising frequency for Bregs in postoperative HCC patients [34]. To our knowledge, Bregs interact with PD-L1 and lead to T cell dysfunction in an IL-10 dependent manner in hepatoma [53], They also accelerate the proliferation and invasion of HCC cells via the CD40/CD154 pathway, and the Breg frequency was positively relating to advanced HCC stages [33].

Increased TIM − 1^+^ Breg cell frequency was closely associated with HCC malignant progression and poor prognosis, which evidently proved to be mediated by tumor sourced high mobility group box 1 (HMGB1) via toll like receptor 2/4 (TLR2/4)-mitogen activated protein kinase (MAPK) pathway. Together with anti-PD-1/PD-L1 therapy against PD-1^hi^Bregs, therapies targeted at Breg specific tags enlighten potential blockage paths against immune escape in HCC [53–55]. Trials on Bregs targeted therapy, like Total glucoside of paeony, was also confirmed with efficiency in HCC rats [56]. However, to fill the gaps between theoretical researches and clinical practices of Bregs targeted treatment against PLC, further explorations on the correlation between Bregs and liver cancer are still needed.

### Passive cellular immunotherapy in primary liver cancer

#### Lymphoid cell based immunotherapy for primary liver cancer

##### Engineering T lymphocytes for primary liver cancer therapy

Originating from myeloid lymphatic stem cells and matured in thymus, T cells participate in many aspects of acquired immunity, both cellular immunity and a lesser extent to humoral immunity, to maintain homoeostasis. CD3^−^CD4^−^CD8^−^ triple negative T cells, namely bone marrow T progenitors, are selected and rearranged for specific T cell receptor (TCR), then differentiate into CD3^+^CD4^+^CD8^−^ αβTCR helper T cells (CD4^+^ T), CD3^+^CD4^−^CD8^+^ αβTCR cytotoxic T cells (CD8^+^ T) and CD3^+^CD4^−^CD8^−^ γδTCR T cells (γδ T) for immune function by thymopoiesis [57]. Dysfunctions of T cells including cell repertoire distributional aberrance, transcriptional regulation and pathway regulative changes, were found to be liable for various kinds of tumorigenesis, and higher proportion of CD3^+^ or CD8^+^ T cells were validated to have correlation with better clinical outcomes in both HCC and iCCA [58–60], therefore T cell targeted intervenes are concerned as potential immune therapeutic strategies for PLC. Gene modified therapy, such as retrovirus transduced epidermal growth factor receptor (EGFR) expressing CD8^+^ T cell, was validated of having tumor growth suppression efficiency in mice [61]. Targeted at blocking up CD8^+^ T cell exhaustion correlated negative costimulatory molecules, immune checkpoint blockages such as tremelimumab, pembrolizumab, nivolumab and ipilimumab are undergoing clinical trials for HCC therapy [62], and etiology specific immunotherapies potentially elicit better outcomes [63]. Clinical case reported that allogenic γδ T therapy enhanced the peripheral immune response against iCCA and improved the patient’s prognosis (NCT02425735) [64].

Among multifarious immunotherapies, genetically TCR engineered T cells (TCR-T) and chimeric antibody receptor engineered T Cells (CAR-T) are pioneer and efficient attempts for the application of engineered T cells in adoptive cellular therapy for PLC. To evaluate the safety and efficiency of TCR-T/CAR-T for solid malignancies, including hepatoma, phase I/II clinical trials are projected and under recruiting (NCT03941626, NCT03638206). Compared with CAR-T, TCR-T is deficient in major histocompatibility complex (MHC) restriction on recognizing TSAs/TAAs but have broader scope on recognizing tumor intracellular proteins, which makes it much advantageous on solid tumor therapy [65]. A-fetoprotein (AFP) specific CD8^+^ T cell clusters, deprived from human leukocyte antigen (HLA)-A2 transgenic AAD mice, were hybridized to generate CD8^+^ T cell with HLA-A2/AFP identifiable TCR, and the hybridoma T cell clones were detected to have effective toxicity on HCC tumor cells [66]. The immune therapeutic potency of HLA-2/AFP specific TCR against HCC was also confirmed with human peripheral blood mononuclear cell (PBMC) derived CD8^+^ T cells [67]. Further trials for AFP specific TCR T cells used on clinical therapy are underway (Table. 2). C-TCR055, AFP specific TCR T cell injection, was selected for function and safety from TCR profiles, and has been used to initiate phase I clinical trials for unresectable HCC therapy (NCT03971747, NCT04368182) [73]. Autologous genetically modified AFP^c332^T cells used for therapeutic trial on advanced HCC are under recruiting (NCT03132792). High affinity purposed TCR engineering, targeted at hepatitis B virus (HBV) [74] and hepatitis C virus (HCV) [75] improved the sensitivity and cytotoxicity of T cell therapy for virus related HCC. HBV specific TCR engineered T cells exhibited cytotoxicity against HBV DNA naturally integrated HCC cells in vitro [76]. Phase I clinical trials tentatively verified that autologous HBV-TCR T cell therapy decreased the pulmonary metastases of HCC free for affecting liver function [68], and provide valuable prevention against HCC relapse with at least 4 weeks’ post-transfer exhibition in patient [69]. New approaches for TSAs/TAAs targeted CAR-T are also springing up in PLC immunotherapy. Glypican-3 (GPC3) targeted CAR-T therapy, such as G3-28Z-41BBL CAR-T [77] and 32A9 CAR-T [78], displayed the cytolytic activities against GPC3^+^ HCC cells. Further optimizations on GPC3-CAR-T positively support its application in HCC treatment. For example, co-expression with IL15/IL21 expanded the antitumor activity of GPC3-CAR-T against HCC at laboratory level [79]. The modification of C-X-C motif chemokine receptor 2 (CXCR2) expression to GPC3-CAR-T promoted its migration and cytotoxicity against HCC cells in mice [80]. The combination with subpharmacologic dose of sorafenib enhanced the antitumor efficiency of GPC3-CAR-T in HCC mouse model [81]. The split GPC3-CAR-T suppressed tumor growth also reduced the risk of severe cytokine release syndrome in vitro and xenograft mice model [82]. GPC3-CAR-T also showed tumor eliminating capabilities in HCC patient derived xenograft (PDX) models (NCT03198546) as a potential CAR-T candidate for PLC therapy [70]. The efficacy, safety and pharmacological properties of GPC3-CAR-T are undergoing clinical trials for further verification (NCT04121273, NCT03884751, NCT03302403, NCT03980288, NCT02905188). Gene modifications for CARs have been constructed to recognize abnormally expressed antigens in malignant cells. The NK group 2 member D (NKG2D) ligands (NKG2DL), highly expressed in tumor cells, were tested as CAR-T targets, and showed that NKG2D-based CAR-T effectively kill NKG2D^high^ HCC cells [83]. Intratumoral medication of AFP-CAR-T was reported to lyse HCC cells via cytokine dependent manner and suppress tumor growth in mouse model [84]. Analogously, CD44-CAR-T [85] and EGFRvIII-CAR-T [86] both released higher levels of cytokines such as INF-γ, TNF-α and better suppressed HCC growth compared with normal/mock T group in vitro and *vivo*. In a phase II clinical trial, patients with advanced HCC received CD133-CAR-T cell infusion after prior systemic therapy, and emerged with 12 months median overall survival (OS) and 6.8 months progression free survival (PFS) [71]. These observations were correlated with significantly increased vascular endothelial growth factor (VEGF) and stromal cell derived factor 1 (SDF-1) levels (both positive for longer OS and PFS), and decreased endothelial progenitor cell (EPC) levels (positive to shorter OS) (NCT02541370). Cocktail treatment, comprised with VEGF-CAR-T, PD-1 monoclonal antibody and CD133-CAR-T, was reported to be effective for iCCA, with a clinical case showed the patient acquired a total of 13-month partial response (PR) from CAR-T therapy, while the toxicities need further exploration [72]. More comprehensive engineered T cell therapy studies are required for further clinical applications.

**Table 2:** Clinical therapeutic trials for engineered T cells against PLC

T cell engineering category	Trial Phase			Enrolled patients backgrounds			Trial information			Reference
	I	II	III	Number.	Location	intervention	Antigen	comments	Identifier	
TCR-T				7/ NCT04677088 10/ NCT02686372 10/ NCT02719782	China	HBV antigen specific TCR T cells	HBsAg	Safety; pulmonary metastases of HCC decreased; valuable prophylaxis against HCC relapse provided	NCT04677088 NCT02686372 NCT02719782	[68, 69]
CAR-T				30	China	GPC3-CAR-T cells	GPC3	HCC cells were eradicared and tumor growth was efficiently suppressed in PDX model	NCT03198546	[70]
				20	China	HCC: CD133-CAR-T cells iCCA: Cocktail treatment comprised with VEGF-CAR-T, PD-1 monoclonal antibody and CD133-CAR-T	CD133 VEGF	Clinical outcomes of dvanced HCC patients improved with manageable safety profile by CD133-CAR-T; Advanced iCCA patient acquired 8.5-month and 4.5-month partial response from VEGF-CAR-T and CD133-CAR-T, respectively.	NCT02541370	[71, 72]

Abbreviations for the table: *TCR-T* T cell receptor engineered T cells, *CAR-T* Chimeric antibody receptor engineered T Cells, *GPC3* Glypican-3, *CD133* Cluster of differentiation 133, *VEGF* Vascular endothelial growth factor, *PD-1* Programmed cell death-1, *HCC* Hepatocellular carcinoma, *iCCA* Intrahepatic cholangiocarcinoma

##### B lymphocytes targeted strategies for liver cancer therapy

B cells originate from lymphoid stem cells and develop into functional subgroups, such as CD5^+^B-1 for inherent immunity, CD5^−^B-2 for adaptive humoral immunity, and Bregs for immune suppression [87]. B cell dysregulation, such as metabolic dysfunction and subset distribution derangement, may contribute to oncogenesis, therefore therapeutic strategies targeted at correcting dysregulations in B cells are likely to generate beneficial antitumor immunity [88, 89]. Patients with type II diabetes were sighted of high immature/transitional B cell frequencies, which might be liable for the procession of chronic hepatitis C (CHC) to HCC and considered as potential disease predictors for CHC [89]. The correlation between B cell dysregulations, either the metabolic changes or subsets redistributions, and the tumorigenesis of PLC are less clear so far. Elimination of CD20^+^B cells with CD4^+^/CD8^+^ T reserved showed inhibition effects on liver cancer progression in Mdr2^−/−^ mice under liver fibrosis condition [90], while clinical studies revealed that B cells were notably decreased in HCC, and the density of tumor infiltrating CD20^+^B cells was positively correlated with superior survival as well as CD3^+^T cells [91, 92]. Further investigations on interactions between tumor infiltrating B cells and T cells, and to verify whether a compensatory mechanism or species variation exists are needed. CD40, a member of TNF receptors, is broadly expressed on immune cells like DCs, B cells as well as some tumor cells. The agonistic reagents to CD40 showed the activation impacts on antitumor immunity as immunotherapeutic candidates [93]. Compared to single monoclonal antibody (mAb) or chemotherapy groups, the combination of anti-CD40/PD-1 with chemotherapy significantly impaired tumor growth and prolonged survival in advanced iCCA murine model [94]. While study also suggested that agonistic anti-CD40 may impel the maturity of myeloid suppressive cells and result in liver damage in mice [95]. Clinical trial to evaluate the efficiency and tolerability of CD40 antibody CDX-1140 in advanced malignancies including PLC is under recruiting for next step estimation on CD40 antagonists (NCT03329950). In depth studies on B cell dependent therapies for PLC and associated mechanisms are warranted in next stage.

##### Natural killer cell based intervetions for liver cancer therapy

Hematopoietic stem cells derived Natural killer (NK) cells are CD3^−^ lymphocytes which classified as minor excretive CD56^bright^ or major cytocidal CD56^dim^ subsets. NK cells play important parts in innate immunity, regulatory immunity, also protect the body from tumor, virus and parasitic bacterium with no prior antigen sensitization requirement [96]. NK cell abnormality is correlated with immunologic defect, as possible causative to liver disease including viral hepatitis, autoimmune disease and liver cancer [96, 97]. For feedback, tumor microenvironments also have impacts on regulating the function and collaboration of NK cells with other immune cells in PLC [98]. NK phenotype has positive or negative effects on HCC in clinical observations, differently. Study showed that the decreasing of CD160^+^NK cells in intra-HCC tissue lead to worsened disease progression with higher recurrence rates, whereas TGF-β1 blocking intervene can restore the CD160^+^NK cell proportion [99]. CD96^+^ NK cells were notably increased in HCC tumor tissue and linked to poor clinical outcomes, while the blockage of TGF-β1 or CD96-CD155 interaction can rescue the NK cell dysfunction and proposed possible routes for PLC therapy [100]. Similarly, CD11b^−^CD27^−^NK cells were highly infiltrated in tumor tissue of HCC patients, positively correlated with tumor progression and poor prognosis [101].

Strategies targeted at NK cell modulation, such as cytokine intervened and gene modified adoptive NK cell transfer, are forefront attempts for liver cancer [102–106]. IL1α was detected to have promotional effects on the cytotoxicity of NK cells against HCC [107]. IL-12/15/18 trafficked to spontaneous HCC mice model were also found to activate NK cells and lower tumor formation [105]. In iCCA, higher expression level of IFN-γ inducible chemokine C-X-C motif chemokine ligand-9 (CXCL9) was correlated with larger tumor infiltrating NK cells and longer postoperative survival [108]. Gene modifications on NK cells, like CAR-NK proposed their therapeutic potency against liver cancer in laboratory level. GPC3-CAR significantly enhanced the cytotoxicity and cytokine production of NK cells when co-cultured with GPC3^+^ HCC cells [104]. Similarly, cytotoxicity against liver cancer presented expanded effects with NKG2D-CD3ζ-DAP10-CAR activated NK cells [103] and *hIFN-α* transferred NK cell lines [106]. Strategies such as iconographic guidance [109] and carrier optimization [110] for NK cell used in liver cancer therapy also showed preferable antitumor efficiency, but still warrant clinical verifications. Clinical trials on allogeneic NK (NCT03937895, NCT03358849, NCT04162158, NCT02562963), CAR-NK adoptive immunotherapy (NCT02839954) to confirm the efficiency, safety and recurrence prevention role against PLC are awaiting for results.

#### Myeloid cell based immunotherapy for primary liver cancer

##### Monocyte/macrophage and liver cancer immunotherapy

Monocytes are generated from myeloid progenitors in bone marrow and released into circulation, then shaped into different phenotypes at specific tissue microenvironment, and can also polarize to replenish the innate macrophages [111–113]. Monocytes and macrophages were reported to have correlation with the progression of PLC [114–116], from which, the idiographic functional pathways may provide optional target molecules for liver cancer immunotherapy.

High monocyte counts predict worse prognosis for postoperative HCC patients, especially the hepatitis virus B infected group [117]. Low lymphocyte to monocyte ratio is companied with inferior HCC outcomes, with cirrhosis arising, total bilirubin elevation, tumor size enlargement and overall survival reduction [118]. Cohort observation on the linkage between lymphocyte monocyte ratio and HCC outcome has also been designed for clinical trial (NCT03869151). Monocyte subgroups were found to promote liver carcinogenesis by complex interactions with immune cells and particular molecules. Studies showed that hepatic stellate cells shift the monocyte into immunosuppressive phenotypes, namely kinds of myeloid derived suppressor cells (MDSCs), may promote liver injury as well as HCC recurrence and progression [119, 120]. Monocytes suppressed the cytotoxicity of retroviral transduced TCR T cells against hepatitis B virus related HCC via PD-1/PDL-1 signaling in a 3D model [121]. Peritumoral monocytes/macrophages were found to have correlation with intratumoral NK dysfunction via blocking CD48 protein 2B4 receptor on NK cells in advanced HCC [98]. Peritumoral monocytes also induced tumor cell autophagy to invade its edge and allow tumor metastasis in HCC [122]. TIE-2 expressing monocytes (TEMs) were positively correlated with HCC angiogenesis [123] and advanced disease stages with sorafenib therapy, emerging its potency as a novel marker in HCC [124, 125]. Ly6C^+^monocytes augment the *myc* triggered carcinogenesis and liver injury, while the tumor burden and survival of mice model can be rescued after monocytes depletion [114]. In spontaneous HCC mouse model, the deletion of IL-6 in monocytes/kupffer cells resulted in tumor suppression for optional therapeutic object [126]. These cell interaction networks and specific molecules suggest optimizational targets for PLC immunotherapy. Hepatic macrophages play crucial roles in hepatocarcinogenesis, both the positive and negative side with signal induced differential phenotypes to our knowledge, such as negatively releasing tumor promoting cytokines in pre-metastatic niche formation and extravasation, positively eliminating hepatoma cells by phagocytosis in cancer cell arrival stage [127–130]. CD169^+^macrophage subpopulations were found to enhance the cytotoxicity and amplification capability of CD8^+^T cells against HCC under anti-CD3 irritation, and exerted suppressive effects on tumor progression [131]. M2 macrophages were stimulated by hepatoma cells and result in tumor growth and metastases for both HCC and iCCA, therefore the blockage at correlated key molecules could serve as beneficial immunotherapeutic strategies in PLC [115, 132, 133]. M1 macrophage loaded hydrogel treatment significantly accelerated HCC tumor necrosis and decreased the tumor size in mice model [134]. Clinical trial on CAR-macrophages for HER2 overexpressing solid tumors, including HCC, is under recruiting (NCT04660929). For the conversion from laboratory research to patient therapy in next stage need more dependable evidence.

Monocytic MDSCs (M-MDSCs), similar to monocytes in morphology and phenotype, are more immunologic suppressive than the other polymorphonuclear MDSCs (PMN-MDSCs) branch in tumor tissue, with an alternative differentiation to tumor associated macrophages (TAM) other than mature macrophages and DCs [135]. Clinical lines showed that the frequency of M-MDSCs and total MDSCs was positively related with alanine transaminase (ALT), AFP, and HCV viral load, while presented negative correlation with CD8^+^ T cell frequency in HCV-HCC patients [136]. Indeed, study also reported that tumor associated fibroblasts (TAFs) treated monocytes, resembling to CD11b^+^ myeloid cells, possess impairments on T cells which negatively correlated with HCC progression [137]. Thus, M-MDSC targeted therapies have caught the attention of researchers and achieved several progresses in HCC treatment. In fibrotic livers, hepatic stellate cell induced increasing of M-MDSCs was found to promote HCC growth in both patients and mice model via p38 MAPK signalling, while status can be rescued by molecular targeting blockage on this pathway [138]. A traditional chinese decoction therapy of jianpi huayu showed its regulatory potency on facilitating the differentiation of MDSCs into macrophages and DCs in HCC mice model, and alleviated the immunosuppression on CD4^+^ T cells, which unfolded new perspectives on DCs/MDSCs targeted treatment against PLC [139].

##### Granulocytes and liver cancer immunotherapy

Granulocytes, mainly composed of neutrophils, eosinophils and basophils, are important components of myeloid cells, which exhibit characterized heterogeneity in inflammation and tumorigenesis [140, 141]. Granulocytic myeloid derived suppressor cells were demonstrated to rescue the blockage of tumor associated macrophages and promote iCCA progression [142]. Neutrophils play crucial roles in tumor mocroenvironment, and were reported to have close correlation with PLC progression [143–145]. High neutrophil counts revealed the predictive capacity to inferior clinical outcomes [145]. The neutrophil to lymphocyte ratio were also positively associated with HCC malignancy, like tumor aggression, extrahepatic recurrence and shrunken overall survival [146, 147]. The neutrophil extracellular traps were found to have tumor promoting effects on nonalcoholic steatohepatitis [144], suggesting that rational elimination or blockage on neutrophils could generate pleasant antitumor effects. Tumor associated neutrophils (TANs) triggered HCC cells and the initiated positive feedback loop for more TANs recruitment result in tumor progression [148]. TANs also recruited both macrophages and Tregs, leading to promote tumor growth and resistance to sorafenib [149]. Granulocytes targeted therapeutic strategies may be effective against PLC, however, more supportive evidence is required.

Morphologically and phenotypically more like neutrophils, PMN-MDSCs present relatively mild immunosuppressive effects but mainly work on regulating tumor specific immune responses, take the dominant place over M-MDSCs in peripheral lymphoid organs [135]. High level of LOX-1^+^ CD15^+^ PMN-MDSCs was proved to have correlation with poor prognosis in HCC patients via T cell suppression, which provided possibilities for PMN-MDSCs targeted therapy in liver cancer [150]. Cell cycle-related kinase (CCRK) depletion leaded suppression of PMN-MDSCs also displayed enhancement on the intratumorous CD8^+^T cells and PD-L1 blockade efficiency against HCC at laboratory level [151]. Further researches are needed to mature the theoretical and practical guideline of PMN-MDSCs targeted treatment against PLC.

#### Cellular immunotherapy combination in primary liver cancer

Treatment options for PLC are typically tailored to disease stages. At early stages of PLC, patients would be appropriate for surgical resection or liver transplantation under certain indications, combined with proper adjuvant therapies, such as Transarterial embolization/TAE, transcatheter arterial chemoembolization/TACE and radiofrequency ablation, to reduce recurrence. For advanced PLC, expectant systemic treatments like cytotoxic chemotherapy, oncolytic virus therapy and immunotherapy are better recommended for patients [11]. Comprehensive therapeutic projects, whether combined with conventional or novel strategies, revealed their superior curative effects against PLC.

Cellular immunotherapy combined with interventional treatment, targeted treatment and radiotherapy were found to have optimized curative effects against PLC. An open label clinical trial enrolled 52 participants on accessing the efficiency and safety of combined treatment against HCC, which composed of TACE and central memory T cells, is completed and awaiting results (NCT03575806). A combination of allogenic NK cell therapy notably increased the median overall survival for patients to 10.1 months, presented synergistic efficiency with irreversible electroporation (IRE) when performed for stage IV HCC [152]. DC-cytokine induced killer (CIK) treatment was detected to improve the antitumor efficiency against liver cancer in rats [153]. Further clinical research showed that a combination of DC-CIK with cryoablation treatment prolong the median overall survival of patients with metastatic HCC compared to single treated groups [154]. In phase I clinical trial, percutaneous microwave ablation prescribed with tumor lysate pulsed DCs, DC-CIK and cytotoxic T lymphocytes showed no adverse effects in HCC patients, and presented effector T cells increasing with Tregs decreasing 1 month after treatment [155]. A combination of toll like receptor-9 agonist and radiofrequency ablation better activated the peripheral blood mononuclear cells against VX2 hepatoma compared with single radiofrequency ablation, increased the antitumor effects and prolonged the survival in VX2 rabbit model [156]. Targeted treatment for blocking tumor progression, either with or without combination to cellular immunotherapy, showed remarkable therapeutic efficiency against PLC. Sorafenib, a multikinase inhibitor, was found to improve the antitumor efficiency in HCC mice model when combined with GPC3-CAR-T [81], and it also ameliorated the outcome of HCC patients when combined with NK cells [157]. AFP specific ET140202-T cells combined with sorafenib or TAE therapy against PLC are under recruiting for a phase I clinical trial (NCT03965546). As fibroblastic growth factor (FGF) signaling was detected to take on crucial parts of cellular characteristics in tumorigenesis, the blocking-up at fibroblastic growth factor receptor (FGFR) has also been focused, such as pan-FGFR inhibitors, and indeed showed its antitumor efficiency in PLC [158–162]. Infigratinib, a pan-FGFR inhibitor, was found to suppress the tumor growth of FGFR^hi^ HCCs possessing sorafenib resistant, and improve the antineoplastic efficiency against HCC either combined with vinorelbine or bevacizumab [163, 164]. Supportively, FGFR mutations are reported to be correlated with indolent iCCA progression [165]. The abundant correlations between FGF signaling and immune cells, known like cell polarization [166] and metabolic regulation [167–169] to macrophage/monocyte, chemotaxis promotion for neutrophil [170], functional cytokine secretory regulation of B cells [171], also provide expectable possibilities for therapeutic combinations of FGFR inhibitors and immune cells against PLC. Lenvatinib, a multi targets inhibitor including FGFR, was proved to prolong the post progression survival of patients with unresectable HCC, and slightly increase the patients’ overall survival compared to first-line sorafenib [172, 173]. Case of iCCA also reported that lenvatinib suppress the metastasis progression when combined with nivolumab, an anti-PD-1 agent, which prompt novel perspectives on FGFR combined immunotherapy by clinic [174]. At laboratory level, Lenvatinib plus PD-1 blockade therapy was found to enhance the potency of effector T cells adjoint with decrease of monocytes and tumor associated macrophages, thus reach a preponderant antitumor effectiveness [175–177]. Further studies are required to insight the combination of immmue cells with multi kinase inhibitors used in PLC therapy. Radiotherapies, such as ^125^I joined with CIK, played inhibitory role on tumor growth in HCC mouse model, and showed improved outcomes [178]. ^125^I combined with NK cell therapy reportedly enhanced immune responses and reduced tumor size in recurrence HCC case [179]. Novel combinations of immune cell therapy were reported to be curative against PLC. Cocktail treatment composed of EGFR-CAR-T and CD133-CAR-T achieved a total of 13-month partial response in an advanced CCA patient [72]. Combination of DCs and CIKs was detected to recover the lung recurrence from liver undifferentiated embryonal sarcoma in one patient [180]. Further studies on the positive and negative impacts of combined cellular immunotherapy treatments are essential for clinical settings.

#### Obstacles and management for cellular immunotherapy in primary liver cancer

Cellular immune therapeutics have inaugurated a new generation of PLC therapies, however, obstacles such as cytokine release syndrome (CRS), loss of response (LOR), and organic adverse events still warrant further research for management [181, 182].

CRS, an overshooting inflammatory response triggered by iatrogenic or pathogenic causes, is the most notable adverse effects companied with immunotherapy and much important factor for evaluating the prognosis [183, 184]. From grade I to IV, CRS ranges a progressive severity of clinical presentations, and the severe multiple organ failure in grade IV can be life threaten [183]. T cell therapies, including CAR-T and other T cell engaged immunotherapies, are major iatrogenic causes for CRS in patients [184, 185]. Studies are sought to manage the CRS toxicity in CAR-T therapy and several approaches have been made to date. Cytokine inhibitors which aimed at blocking CRS in CAR-T treated patients were demonstrated to reduce the CRS toxicities in laboratory level. Itacitinib, a potent selective JAKI inhibitor, was detected to have potency on reducing CRS implicated cytokines in vitro and *vivo*, without suppression on antitumor efficiency. Further phase II clinical trial on validating the prophylaxis of itacitinib against CAR-T correlated CRS are initiated (NCT04071366) [186]. The application of tocilizumab, an antibody against IL6 receptor, also exhibited protective role in patients suffered from grade II to III CAR-T induced CRS [187]. Corticosteroids, continuous renal replacement therapy (CRRT), delivery optimization for immunotherapies and next generation CAR-T (with ON−/OFF-switch components or multiple antigen targetted gates) are also recommended for CRS management [185, 188–190].

Common categories for LOR, such as off-target effects and immune resistance, are blockages eager for solutions in cancer immunotherapy. Targeted at NK cell receptors (NKp46, CD16) and cancer cell antigens, NK cell engagers (NKCEs) reduced the off-target effects and revealed its integrated functions of both IgG antibodies multitude and tumor growth suppression, thus enhancing the antitumor efficiency of NK based immunotherapy [191]. Improved delivery strategies may also elevate precision and reduce the off-target effects for T cell based antitumor therapies [189]. Insufficient infiltration of CD8^+^T, aberrant expression of immune checkpoint molecules, and heterogeneity of individual genome, may all contribute to resistance in immunotherapy [192, 193]. Therefore, strategies to improve the management of therapeutic resistance need further exploration.

Organic adverse events vary from different strategies of immunotherapies and individual specificity, while similarly result in inferior prognosis and curative effects in patients. Complications such as neurotoxicity [194, 195], hepatotoxicity [196], infection [197, 198] and severe cutaneous adverse reactions [199] have been reported in engineered T cell therapy. Steroids are recommended for isolated immune effector cell associated neurotoxicity (ICANS) as first line therapy, while there are distinguished recommendations on managing grade I to IV stages for ICANS, followed the guideline of American society for transplantation and cellular therapy (ASTCT) [198]. For grade I ICANS, support treatment and monitoring are recommended, and for grade II to III, corticosteroids are indicated therapy, while for higher grade of ICANS, ICU guardian and airway protection are necessary in treatment [198, 200]. For hepatotoxicity induced by immune checkpoint inhibitors, corticosteroids are administered for grade II or higher hepatic lesion with symptoms [201]. Prophylaxes against infections, such as herpes simplex (HSV) and *Pneumocystis jirovecii* prophylaxis, are recommended after CAR-T therapy [198]. Further studies on the safety and adequate source of immune cells for PLC therapy are required [198, 202].

## Conclusions

Immune cell based therapy is attractive for PLC treatment, especially the pioneering TCR-T/CAR-T approaches of adoptive cellular therapy. Mentioned as lymphoid or myeloid based cellular therapy, passive immunotherapies share the advantages on relatively unrestricted therapeutic patterns and have reached much progress in PLC therapy. On the other side, active cellular immunotherapy focuses on rebuilding the intrinsic immune microenvironment to exert its antineoplastic potency against PLC, which is superior in the risk reduction of uncertain triggered immune response while more in need of comprehensive considerations on the tumor immunogenicity and host’s immune status. Obstacles for cellular immunotherapy still remain and require preferable solutions when finally applied to PLC patients.

## Data Availability

Not applicable.

## Abbreviations

PLC: Primary liver cancer
HCC: Hepatocellular carcinoma
iCCA: Intrahepatic cholangiocarcinoma
TSAs/TAAs: Tumor-specific/associated antigens
APCs: Antigen presenting cells
DCs: Dendritic cells
GM-CSF: Granulocyte macrophage colony stimulating factor
IL-2: Interleukin-2
Treg: Regulatory T cell
Breg: Regulatory B cell
tTreg cells: Thymus-derived naturally occurring Tregs
pTreg cells: Peripherally derived induced Tregs
CTLA-4: Cytotoxic T lymphocyte associated antigen-4
TNF-α: Tumor necrosis factor-α
TLR-4: Toll like receptor-4
PD-1: Programmed cell death-1
PD-L1: Programmed cell death-ligand 1
TGF-β: Transforming growth factor β
FOXP3: Forkhead box protein P3
GITRL: Ligand to Tregs evoked glucocorticoid induced tumor necrosis factor receptor
HGF: Hepatocyte growth factor
HMGB1: High mobility group box 1
MAPK: Mitogen activated protein kinase
TIM-1^+^: T cell immunoglobulin mucin domain-1 positive
TCR: T cell receptor
CD4^+^ T: CD3^+^CD4^+^CD8^−^ αβTCR helper T cells
CD8^+^ T: CD3^+^CD4^−^CD8^+^ αβTCR cytotoxic T cells
γδ T: CD3^+^CD4^−^CD8^+^ αβTCR cytotoxic T cells
EGFR: Epidermal growth factor receptor
TCR-T: TCR engineered T cells
CAR-T: Chimeric antibody receptor engineered T Cells
MHC: Major histocompatibility complex
AFP: A-fetoprotein
HLA: Human leukocyte antigen
PBMC: Peripheral blood mononuclear cell
HBV: Hepatitis B virus
HCV: Hepatitis C virus
GPC3: Glypican-3
CXCR2: C-X-C motif chemokine receptor 2
PDX: Patient derived xenograft
NKG2D: NK group 2 member D
NKG2DL: NKG2D ligands
EGFRvIII: Epidermal growth factor receptor variant III
INF-γ: Interferon-γ
OS: Overall survival
PFS: Progression free survival
VEGF: Vascular endothelial growth factor
SDF-1: Stromal cell derived factor 1
EPC: Endothelial progenitor cell
PR: Partial response
mAb: Monoclonal antibody
NK cells: Natural killer cells
CXCL9: C-X-C motif chemokine ligand-9
DAP10: DNAX-activating protein 10
hIFN-α: Human interferon-α
MDSCs: Myeloid derived suppressor cells
TEMs: TIE-2 expressing monocytes
TANs: Tumor associated neutrophils
CIK: DC-cytokine induced killer
CRS: Cytokine release syndrome
LOR: Loss of response
CRRT: Continuous renal replacement therapy
NKCEs: NK cell engagers
ICANS: Immune effector cell associated neurotoxicity
ASTCT: American society for transplantation and cellular therapy
HSV: Herpes simplex
FGF: Fibroblastic growth factor
FGFR: Fibroblastic growth factor receptor
M-MDSC: Monocytic MDSCs
PMN-MDSCs: Polymorphonuclear MDSCs
TAM: Tumor associated macrophages
CCRK: Cell cycle-related kinase
